# Charge and Polarity Preferences for *N*-Glycosylation: A Genome-Wide *In Silico* Study and Its Implications Regarding Constitutive Proliferation and Adhesion of Carcinoma Cells

**DOI:** 10.3389/fonc.2018.00029

**Published:** 2018-02-28

**Authors:** Muhammad Ramzan Manwar Hussain, Zeeshan Iqbal, Wajahat M. Qazi, Daniel C. Hoessli

**Affiliations:** ^1^Key Laboratory of Genome Sciences & Information, Beijing Institute of Genomics (CAS), Chinese Academy of Sciences, Beijing, China; ^2^University of Chinese Academy of Sciences, Beijing, China; ^3^Institute of Molecular Sciences & Bioinformatics, Lahore, Pakistan; ^4^Department of Physics, GC University Lahore, Lahore, Pakistan; ^5^Center for Intelligent Machines and Robotics, Department of Computer Science, COMSATS Institute of Information Technology, Lahore, Pakistan; ^6^Panjwani Center for Molecular Medicine and Drug Research, International Center for Chemical and Biological Sciences, University of Karachi, Karachi, Pakistan

**Keywords:** *N*-glycosylation, cancer, human proteins, genome-wide mapping, charge and polarity, EGFR, cadherins, epithelial-to-mesenchymal transition

## Abstract

The structural and functional diversity of the human proteome is mediated by *N*- and *O-*linked glycosylations that define the individual properties of extracellular and membrane-associated proteins. In this study, we utilized different computational tools to perform *in silico* based genome-wide mapping of 1,117 human proteins and unravel the contribution of both penultimate and vicinal amino acids for the asparagine-based, site-specific *N*-glycosylation. Our results correlate the non-canonical involvement of charge and polarity environment of classified amino acids (designated as L, O, A, P, and N groups) in the *N*-glycosylation process, as validated by NetNGlyc predictions, and 130 literature-reported human proteins. From our results, particular charge and polarity combinations of non-polar aliphatic, acidic, basic, and aromatic polar side chain environment of both penultimate and vicinal amino acids were found to promote the *N*-glycosylation process. However, the alteration in side-chain charge and polarity environment of genetic variants, particularly in the vicinity of Asn-containing epitope, may induce constitutive glycosylation (e.g., aberrant glycosylation at preferred and non-preferred sites) of membrane proteins causing constitutive proliferation and triggering epithelial-to-mesenchymal transition. The current genome-wide mapping of 1,117 proteins (2,909 asparagine residues) was used to explore charge- and polarity-based mechanistic constraints in *N*-glycosylation, and discuss alterations of the neoplastic phenotype that can be ascribed to *N*-glycosylation at preferred and non-preferred sites.

## Introduction

Glycosylation of proteins is a most complex form of co- and post-translational modifications introducing structural diversity to proteins in the form of *O*- and *N*- linked sugar moieties (1–8). The covalent addition of complex glycans to the amide side chain of asparagine (*N*-glycosylation) and hydroxyl groups of serine and threonine (*O*-glycosylation) generates a large number of glycoforms that are credited for the modulation of diverse cellular functions (4, 5, 9–11).

Proteins that undergo *N*-linked glycosylation are biosynthesized on membrane-associated ribosomes and their signal peptide is removed by a signal peptidase as they emerge into the lumen of the rough endoplasmic reticulum. In the endoplasmic reticulum (ER), the oligosaccharyl transferase (OT) mediates the co-translational transfer of a lipid-linked tetradecasaccharide (GlcNAc2-Man9-Glc3) from a dolichol phosphate to an asparagine included in a NXS/T sequon. The selective recognition by OT of the consensus sequence (NXS/T) has enabled investigation of the structural requirements for *N*-glycosylation. The rapid increase of substrate data for protein *N*-glycosylation has led to the development of different databases and prediction tools: dbPTMs, UniProt, NetNGlyc and MAPRes (Mining Association Patterns among preferred amino acid residues in the vicinity of amino acids targeted for post-translational modifications) (10, 12–15).

Human proteins including growth factors, growth factor receptors, cell-surface proteins and secretory proteins are among the substrates that are *N*-glycosylated to perform key biological functions (16–27). The statistical analysis of the sequence contexts for *N*-glycosylation (preferred and non-preferred motifs) is needed to explore the biological relationships between sequence, structure, and function of glycoproteins. MAPRes is a valuable tool to define the significantly preferred and non-preferred amino acids in the vicinity of a *N*-glycosylation site by resorting to the association rule mining technique (12, 28). The association pattern/rule is established between two or more frequently occurring entities that are in correlation. The new version of MAPRes has the capacity to analyze the sequence environment of the modified residues according to the biophysical and biochemical properties (polarity and charge) of the amino acids. NetNGlyc^1^ is another important computational tool that predicts the *N*-glycosylation (N+) and non-*N*-glycosylation (N^−^) sites on the basis of potential score and consensus sequences within the target protein (29).

In this study, we have identified 2,909 *N*-glycosylated sites (N+ sites) in 1,117 human proteins in which the majority (96.5%) of N + sites is followed by the canonical motif of NXS/TY. According to our MAPRes analysis for general protein sequence analyses, Val at +1, Ser/Thr at 2, Leu/Val at 3 and Leu at −5 positions were found significantly preferred residues to mediate the glycosylation of Asn residues in the human proteome. After classifying amino acids’ charge and polarity according to properties of their side-chain R-groups, significant preference for *N*-glycosylation was found for non-polar, uncharged R-groups (Leu/Val/Gly/Ala/Ile: O) at position 1, polar R-group (Met/Thr/Ser/Cys/Asn/Gln: L) at position 2, polar, negatively charged acidic R-groups (Asp, Glu: N) at position 3/5/−4 and aromatic amino acids: Phe/Trp/Tyr: A, at position 3/−5/−1. Furthermore, we validated the MAPRes-predicted preferred association pattern for the wider N-glycosylation sequence contexts by using the NetNGlyc 1.0 server and 130 literature-reported UniProt proteins, and provided further evidence that charge and polarity of O amino acids (Gly/Ala/Val/Leu/Ile) at position 1, A amino acids (Phe/Trp/Tyr) at positions −6, −5, −2, −1,1,3, and 10, P-amino acids (Lys/Arg/His) at positions −9, −3, 9, 10, and N amino acids (Asp/Glu) at positions −4/3/5, in combination with L amino acids at position 2, is likely to generate significantly preferable environments for the N-glycosylation of human proteins. Any change in charge and polarity environments may, therefore, result in aberrant N-glycosylation on both preferred and non-preferred sites.

## Materials and Methods

As a first step, preferred amino acids and association patterns were found around *N*-glycosylated (N+) and non-*N*-glycosylated (N−) residues by using MAPRes. Then, preferred amino acids and association patterns were determined on the basis of polarity and charge of the surrounding amino acids of N+ and N− residues. Next, association patterns mined by MAPRes were validated with the NetNGlyc 1.0 server (see text footnote 1) for 15 biologically important proteins. The second level of validation was performed by using 130 literature-reported human proteins with 438 *N*-glycosylated motifs from the UniProt database.^2^

### Data Assembly

The primary sequences for *N*-glycosylated proteins were downloaded from dbPTM, version 3.0,^3^ which is a database containing information about phosphorylation and glycosylation. The downloaded data for *N*-glycosylation included 16,915 entries in total and provided all required information for MAPRes analysis except for the protein primary sequences (from UniProt), which were added manually in the final dataset. All entries other than human proteins were removed. In the final dataset, only 1,117 proteins and 2,909 asparagine residues were found modified.

### Data Cleaning

The data cleaning of the final dataset was essential for estimating modified residues on vicinal amino acids. Several types of errors that can be generated during the compilation of the dataset such as incorrect position of the modified residues, presence of non-standard characters in the sequence, incorrect sequence length and repetitions of entries. These inconsistencies were identified and removed by utilizing the Data Inconsistency and Duplication Check module of MAPRes.

### Dataset

To the environments of the N+ (positive) and N− (negative), the negative sites were annotated in the final dataset. There were in total 31,052 negative sites (non-modified Asn) in the final dataset of human *N*-glycosylation proteins (Table 1). The ratio of the negative to positive sites is a very high number which could bias the results. To balance the number of negative and positive sites, a computational module was utilized, which selects the random entries in any dataset and can balance data one to one (1:1) or one to two (1:2) positive to negative sites. In this study, the positive to negative sites were analyzed on a 1 to 1 basis.

**Table 1:** Statistics of assembled data and MAPRes output.

Observations	Glycosylated		Non-Glycosylated	
	General protein dataset	Encoded protein dataset	General protein dataset	Encoded protein dataset
Total number of proteins	1,117			
Number of targeted sites	2,909		31,052	
Number of significantly preferred sites	43	16	32	8
Number of association patterns	23	39	18	17
Number of unique association patterns	8	24	17	9
Support level (%)	5–55	5–95	5–10	5–30

### Classification of the Amino Acids

The new version of MAPRes can mine association patterns for neighboring amino acids of modified residues on the basis of their specific biophysical and biochemical properties. In this study, the association patterns mined on the basis of polarity and charge of the amino acids were distributed into five different groups (Table 2). Specific letters were used to substitute the symbols of standard amino acids such that positively charged R-group amino acids were replaced with P, negatively charged R-group with N, polar, uncharged R-group with L, non-polar, aliphatic R-group with O, and aromatic R-group amino acids with A (Table 2).

**Table 2:** Classification of for charge and polarity specific analysis of amino acids.

Chemical nature of side chain	Amino acids	Abbreviation
Positively charged R-groups	K, R, H	P
Negatively charged R-groups	D, E	N
Polar, uncharged R-groups	S, T, C, M, N, Q	L
Non-polar, aliphatic R-groups	G, A, V, L, I	O
Aromatic R-groups	F, Y, W	A

### Preference Estimation and Association Rules Mining

After the classification of the amino acids according to the polarity and charge, the whole dataset was further divided into *N*-glycosylated and non-*N*-glycosylated datasets. MAPRes supplied the charge and polarity information to estimate the significantly preferred amino acids and association rule mining for neighboring amino acids for all datasets (Table 3). In the first step, MAPRes generated 21 amino acid long peptides (Asn at 0 position and 10 amino acids on each side) from the protein dataset and estimated their preference (preferred amino acids corresponding to N+ and N− residues). Next, association rules/patterns were estimated by utilizing the preferred amino acids and their correlation with the modified residues. Association analyses were carried out at different possible support values for all datasets. There are four datasets in total, two for primary sequence of proteins (one is *N*-glycosylated “N+” and other is non-*N*-glycosylated “N−”) and two for classified/encoded sequence of proteins (*N*-glycosylated and non-*N*-glycosylated) for which MAPRes mined association rules (Table 4).

**Table 3:** Significantly preferred sites around *N*-Glycosylated and non-*N*-Glycosylated residues for general protein sequence analyses.

Amino Acids	*N*-Glycosylated residues	Non-*N*-Glycosylated residues
**Significantly preferred positions for general protein dataset**		

C	−10, 1, 7	−2, 7, 9
D	−6, 5, 9	
E	−4, 3, 5	−7, −1
F	−5, −2, −1, 10	10
G	1	
H	−3, −2	
I	1, 4, 6	−4, −3, −2, 1, 2, 4, 5, 7
K	−3, 9, 10	4, 8
L	−5, 3	−3
N	−10, 8, 9, 10	−10, −7, −6, −1, 1, 7, 8
Q	−9,−8,−1, 10	−1
R	−7	−1
S	1, 2	
T	2	−5, 2
V	1, 3, 5	
W	3,	
Y	−2, −1, 1	−6, −5, −1, 3

**Significantly preferred positions for encoded protein dataset**		

A	−6, −5, −2, −1, 1, 3, 10	−6, −3, −2, 3, 10
L	2	−7, 2
N	−4, 3, 5	–
O	1	–
P	−9, −3, 9, 10	8

**Table 4:** Association rules/patterns mined by MAPRes for *N*-Glycosylated residues for general and classified protein sequences.

Association patterns	Confidence level (%)	Support level (%)
**Rules for general protein dataset**		

<L,−5><T,2>	100	5
<T,2><L,3>	100	5
<V,1><T,2>	100	5
<T,2><V,3>	100	5
<L,−5>	100	10
<L,3>	100	10
<S,2>	100	10, 15, 20, 25, 30, 35, 40
<T,2>	85.87	10, 15, 20, 25, 30, 35, 40, 45, 50, 55

**Rules for encoded protein dataset**		

<A,−5><O,1><L,2>	100	5
<A,−2><O,1><L,2>	100	5
<N,−4><O,1><L,2>	100	5
<O,1><L,2> <N,3>	100	5
<O,1><L,2><N,5>	100	5
<P,−9><O,1><L,2>	100	5
<P,−3><O,1><L,2>	100	5
<O,1><L,2><P,9>	100	5
<O,1><L,2><P,10>	100	5
<A,−5><L,2>	100	10
<A,−2><L,2>	77.25	10
<A,−1><L,2>	100	10
<L,2><A,3>	74.88	10
<L,2><A,10>	77.31	10
<N,−4><L,2>	100	10
<L,2><N,3>	100	10
<L,2> <N,5>	100	10
<P,−9><L,2>	100	10
<P,−3><L,2>	100	10
<L,2><P,9>	100	10
<L,2> <P,10>	100	10
<O,1><L,2>	100	15, 20, 25, 30, 35, 40
<L,2>	76.12	45, 50, 55, 60, 65, 70, 75, 80, 85, 90, 95

### Validity of Association Rules

The validity of the association patterns/rules were performed by the NetNGlyc 1.0 Server for the *N*-glycosylation and the 130 UniProt-reviewed human proteins comprising 438 *N*-glycosylated motifs (Table 5). At the first level of validation, the FASTA sequences of 15 biologically important (randomly selected) proteins were retrieved from UniProt. The modification potential for different residues was found by utilizing the NetNGlyc 1.0 Server (see text footnote 1). The 21 amino-acid long peptide assigned Asn at positions 0 and 10 amino acids on each side in the datasets of both predicted and non-predicted sites. The association patterns mined by MAPRes for N+ and N− were searched in the predicted and non-predicted peptide datasets. The percentage of peptides consistent with the association patterns was calculated. The same procedure was applied to the association rules found on the basis of the classification of the amino acids. At this step, the predicted and non-predicted peptide datasets were encoded according to the previously defined classification of the amino acids. In the second validation step, 438 N-glycosylated motifs from 130 literature-reported proteins were analyzed and the percentage of N-glycosylated motifs validating the MAPRes association patterns was calculated. The protein modeling was performed by using Iterative Threading ASSEmbly Refinement (I-TASSER) (30).

**Table 5:** Validation of association patterns mined by MAPRes with the NetNGlyc prediction model.

	Predicted	Non-Predicted
Total no of residues (Asn)	229	642
Number of peptides in which rules were found	216	378
Number of encoded peptides in which rules were found	195	397
Percentage of conformity with patterns mined by MAPRes (%)	94	59
Percentage of conformity with patterns mined by MAPRes for encoded dataset (%)	85	62

## Results

### Rules Mined by MAPRes for N+ Sites

**Figure 1:**
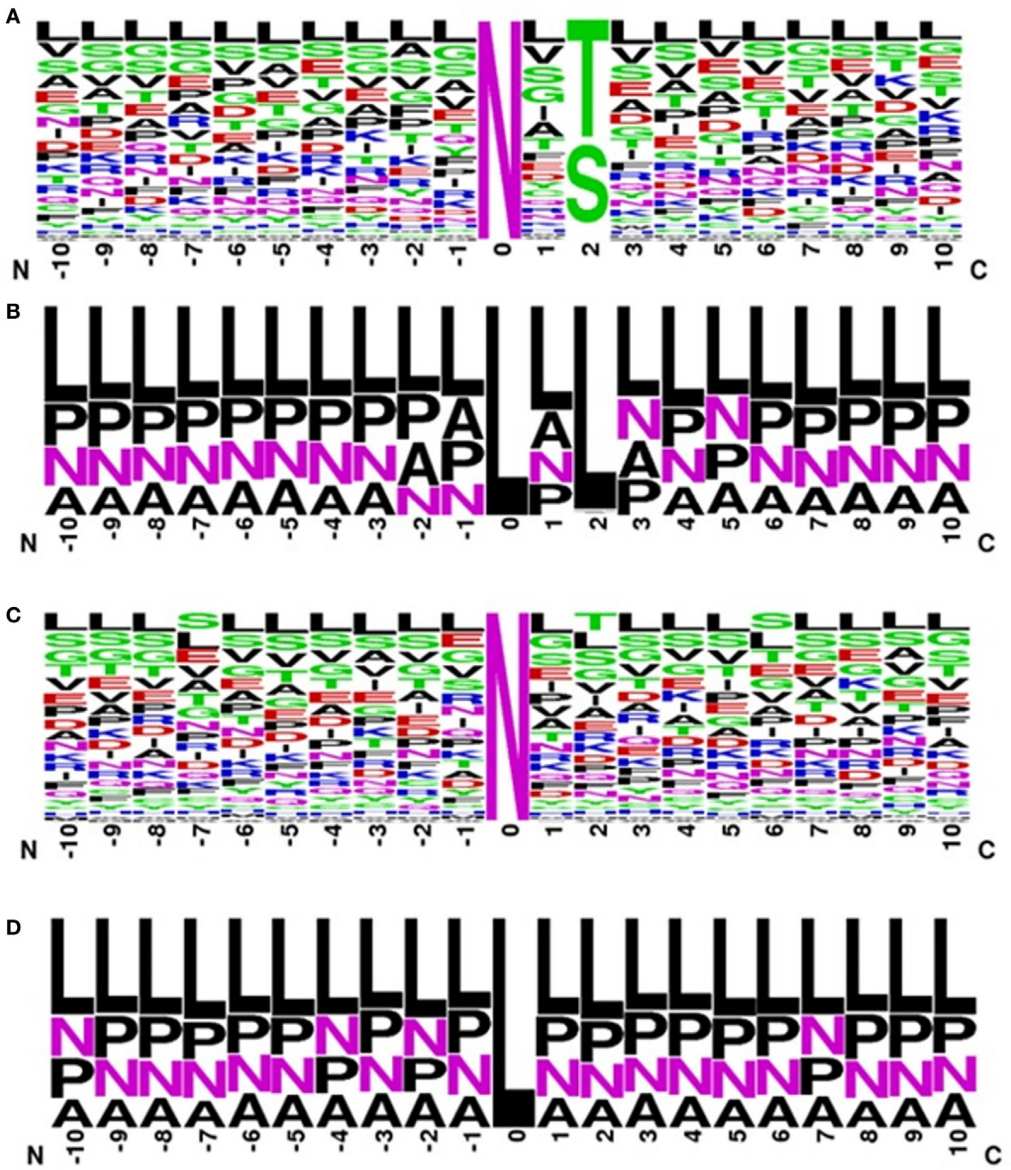
Frequency logo for sequence environment of N + residues for **(A)** general dataset, **(B)** classified dataset and of N- residues for **(C)** general dataset **(D)** classified dataset.

MAPRes identified 43 significantly preferred amino acid positions around *N*-glycosylated residues (Table 1). The Phe, Asn, and Gln were found preferred at four different positions and Asp, Gly, Ile, Lys, Val, and Tyr at three positions (Table 3). MAPRes mined association patterns on the basis of these significantly preferred amino acids allowed to develop a correlation between significantly preferred amino acids and modified residues. MAPRes suggested 23 association patterns that were the sum of all patterns mined at different support levels. After removal of repeated patterns/rules found at different support levels, only 8 distinctive rules for *N*-glycosylated residues were retained. From those patterns, Thr and Ser at position 2 (Figure 1A) were found at the highest support levels. MAPRes also found some other residues about which a correlation could be developed with *N*-glycosylated residues with Leu at positions −5/3 and Val at positions 1 and 3. Seven out of eight association patterns mined at 100% confidence level (Table 4).

In case of classified amino acids, MAPRes found 16 significantly preferred sites for *N*-glycosylated residues and 24 unique association patterns. The A amino acids were found significantly preferred at 7 different positions. Similarly, L, O, A, P, and N amino acids were also found preferred at several positions (Table 3). The range of confidence levels was 74.88–100%. The range of support levels was from 5 to 95%. The L amino acids at position 2 (Figure 1B) were found at the highest support level (95%). Another rule found at multiple support level was <O,1> <L,2> (Figure 1B) and remaining association patterns were mined at only 5 and 10% support levels (Table 4).

### Rules Mined by MAPRes for N-Sites

MAPRes also mined association rules for N-residues at different support levels. There were 32 significantly preferred sites identified by MAPRes for N-sites (Table 2). Moreover, 18 association patterns in total were mined by MAPRes for N-sites and only one pattern was found at multiple support levels. Lys at position −3 (Figure 1C) was the only residue which was mined at 10% support level. The Ile and Asn were found significantly preferred at 8 and 7 different positions, respectively, and all of these positions were also correlated with N-sites. Tyr was also found preferred at four different positions but none of these developed a correlation with N-residues. The range of confidence level was 14.13–100% for N-residues. The <T,2> (Thr at position +2) had the lowest confidence level (14.13%).

The results concerning the significantly preferred positions and association patterns on the basis of polarity and charge of the surrounding amino acids indicated that the A amino acids were highly preferred at five positions (Table 3). L amino acids were also preferred at two positions and all of these preferred residues were found in association patterns for N-sites. The two association patterns <L,−7> and <L,2> (Figure 1D) were found at multiple support levels. The range of the confidence levels was 23.88–100% for the association patterns mined by MAPRes for classified dataset of non-*N*-glycosylation.

### Validation of the Association Patterns Mined by MAPRes

**Figure 2:**
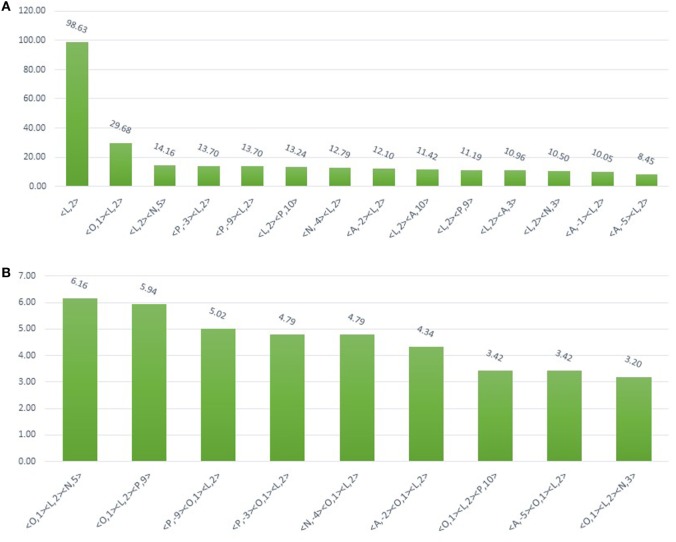
Bar plot showing the 438 *N*-glycosylated residues from 130 UniProt-reviewed proteins validating the MAPRes association rule for preferred *N*-glycosylation sites. **(A)** From 438 *N*-glycosylated motifs of 130 proteins, based on charge and polarity preference, 99% of *N*-glycosylated residues were found with L-group at 2 (<L,2>) indicating the significant preference for N-glycosylation. The other classified groups (O, N, P, and A) were found in charge and polarity combination with L at 2. For instance, 30% *N*-glycosylated motifs were observed with O at 1 and L at 2 (<O,1><L,2>), 14% N at position 5 (<L,2> <N,5>), 13% P (<L,2> <P,10 >; <P,−9><L,2>; < P,−3> <L,2>), and 12% A at −2<A,−2><L,2>. **(B)** Three groups combination (<O,1><L,2><N,5>; <O,1><L,2><P,9>; <P,−9><O,1> <L,2>; <P,−3><O,1><L,2>; <N,−4><O,1><L,2>; <A,−2><O,1> <L,2>; <O,1><L,2> < P,10>; <A,−5><O,1><L,2>; <O,1><L,2><N,3>) were ranging from 6 to 3% motifs.

**Figure 3:**
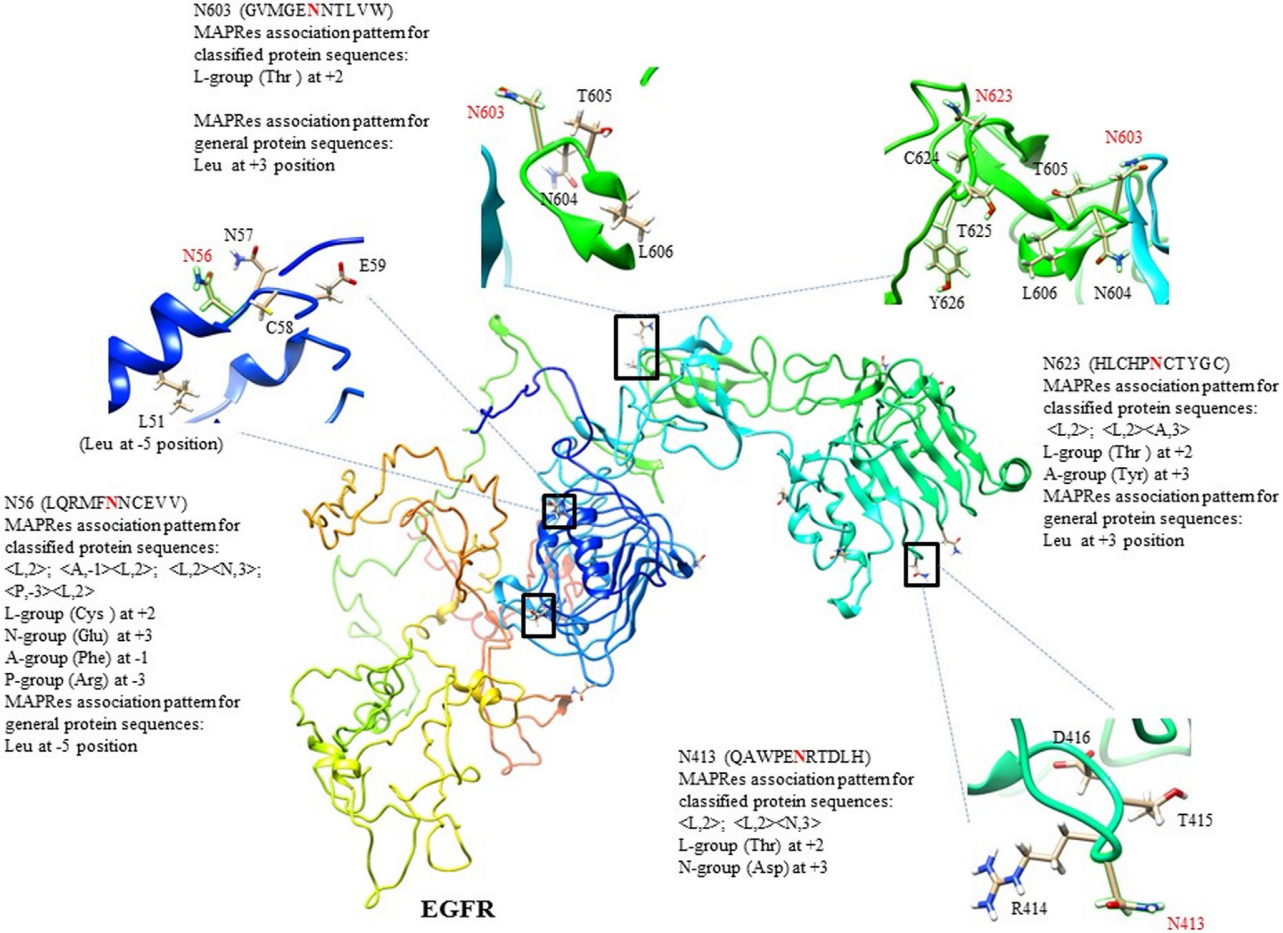
3D-structure of human EGFR with C-score = −2.91, and modeling of selected *N*-glycosylated motifs from 13 sites following the MAPRes association pattern as NXT/S/CY sequon.

**Figure 4:**
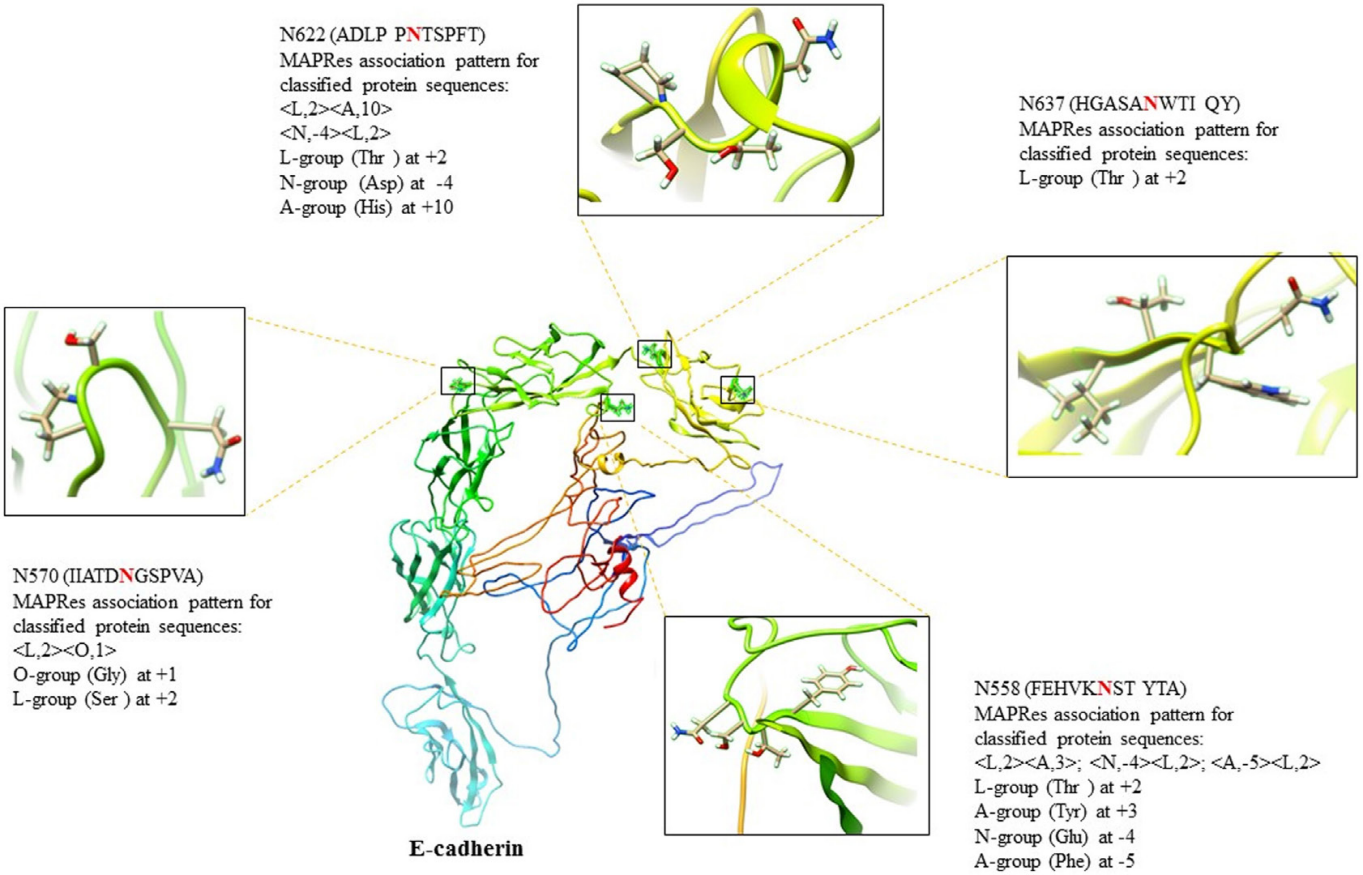
E-cadherin (E-cad) architecture (C-score = −2.49) with N558, N570, N622, and N637 amino acids chemistry validating the MAPRes association pattern with the NXS/TY sequon.

In the first level of validation, the association patterns mined by MAPRes for *N*-glycosylated and non-*N*-glycosylated residues were determined by utilizing the NetNGlyc server. There were 226 potential sites for *N*-glycosylation (N+) found by NetNGlyc from the selected 15 proteins. The rest of the 642 Asn were considered to be N-residues. Peptides of 21 amino acids were generated for both N+ and N− residues and patterns mined by MAPRes for *N*-glycosylated and non-*N*-glycosylated, and found in both datasets. The confirmatory percentage for association patterns in the peptide dataset was found to be 94% for the *N*-glycosylated dataset and 59% for the non-glycosylated dataset (Table 5). As for the validation of the association patterns mined by the MAPRes on the basis of polarity and charge, an explicit technique was used. Namely, the amino acids of the predicted and non-predicted dataset were decoded according to the defined classification of the amino acids, and association patterns searched in this dataset. This validation procedure for encoded datasets provided a good percentage of conformity, with 85% for the *N*-glycosylated and 62% for the non-*N*-glycosylated sites (Table 5). In the second level of validation, the 438 *N*-glycosylated motifs from 130 experimentally known proteins were retrieved from UniProt (reviewed human proteins) and sequence-based statistical analysis of *N*-glycosylated motifs validated the association rules of MAPRes. From the general dataset, around 99% of *N*-glycosylated motifs were found in the NXS/TY sequon (40% of *N*-glycosylated motifs were found in the NXSY sequon, 59% in the NXTY sequon). From classified datasets, among 438 residues, 99% were L amino acids at position 2 (<L,2>), and 30% of total were O-amino acids at position 1, in combination with L amino acids at position 2, to validate the <O,1><L,2> rule. Therefore, a strong correlation was observed between polar and non-polar R-groups in the NXS/TY sequon. The presence of O amino acids (G, A, V, I, L) at position 1 and of L amino acids at position 2 were characteristic for *N*-glycosylation (Figure 2A). From 99% *N*-glycosylated motifs with L amino acids at position 2, 14%, 12% and 10% residues were occupied with amino acids containing negatively charged polar R-group (N amino acids: Asp/Glu) at 5, −4, and 3 positions. In addition to O and N amino acids, A amino acids also showed preference with at −2, 10, 3, −1, and −5 positions, in combination with L amino acids at 2 (Figure 2A). The presence of aromatic amino acids penultimate or vicinal to the *N*-glycosylated site supports the *N*-glycosylation mechanism in the presence of Cyst/Ser/Thr at 2. The P-amino acids, in combination with L at 2, occupied 11 to 13% of positions −3, −9, and 9. Hence, the particular combination of non-polar, polar, aromatic, positive, and negatively charged residues in the vicinity of Asn supports the *N*-glycosylation process (Figure 2). For instance, in EGFR, an important oncogenic driver in various carcinoma, the MAPRes association patterns for classified protein sequences validated L (Thr/Ser/Cys) at position 2 (Figure 3). Similarly, the occupancy by O amino acids at position 1, N at 3/−4, at −1/−2/3/10, and P at −3/9/10 was validated by the MAPRes association pattern in 13 *N*-glycosylated motifs [N56 (NNCE), N73 (NYDL), N128 (NKTG), N175 (NMSM), N196 (NGSC), N352 (NATN), N361 (NCTS), N413 (NRTD), N444 (NITS), N528 (NVSR), N568 (NITC), and N603 (NNTL)], localized in the extracellular EGFR (Figure 3), suggesting the preference of non-polar aliphatic, negatively charged polar, positively charged polar, and aromatic amino acids in combination of L-group amino acids for *N*-glycosylation in EGFR. In addition to EGFR, in E-cadherin (E-cad), another key regulator of neoplastic cells, the extracellular *N*-glycosylation sites “N558 (NSTY), N570 (NGSP), N622 (NTSP), N637 (NWTI)” were modeled to validate the MAPRes association pattern (Figure 4).

## Discussion

Different tools and techniques have been developed to understand the decisive roles of glycoconjugates in biological systems (3, 4, 6, 7, 10, 18, 27, 31–34). However, many important facets of glycosylation in protein function remain to be explained.

*N*-glycosylation is a biologically relevant protein modification for the regulation of signaling, protein–protein interactions and protein folding and stability (6, 31, 35, 36). *N*-glycosylation is initiated in the ER and modified within the Golgi stacks, with the removal of mannose residues and sequential addition of GlcNAc and fucose. However, during passage through the Golgi, *N*-glycosylation depends both upon the structure of the protein as well as the amount and quality of processing enzymes available (31, 36).

Based on the hypothesis that the nature of both penultimate and vicinal amino acids contributes to the regulation of site-specific glycosylation, different computational tools have been developed to unravel the upstream and downstream characteristics of glycosylation sites (12, 37). Such computational tools should be useful in screening glycosylation sites in oncologically relevant proteins from cancer tissues (12, 38, 39). MAPRes is one of such computational tools that estimates significantly preferred sites around modified residues and mines association rules/patterns (10). The preference estimation of significantly preferred sites and association pattern-mining at various support levels has already been studied extensively for large amounts of data (38).

In this study, MAPRes supports the Ser and Thr at position 2 at the highest level compared to the other mined patterns (Table 4). At the level of charge and polarity, amino acids with polar-uncharged R-groups encoded in as L residues at 2 position showed the highest support level (95%) for *N*-glycosylation. In other studies, the same motif was identified by the presence of Ser/Thr at position 2 around glycosylated Asn that can affect the polarity of the protein (39–41). Our results are consistent with a recent study stating that the Asn is preferably glycosylated when position 2 is occupied by Ser/Thr, and provided position 1 is not Pro (42). In addition, our results also highlight the marked preference for Thr at position 2 instead of Ser in NXS/T motif which is also consistent with the literature (40, 41). The combined effects of both O amino acids, carrying non-polar aliphatic R-groups, at position 1, and L amino acids with polar R-groups at position 2, were found to enhance *N*-glycosylation, as examplified by the rule <A,−1> <L,2> (Figure 2A). Hence, to support *N*-glycosylation, non-polar R-group containing O amino acids were found sandwiched between polar R-groups containing Asn and Cys/Ser/Thr. Moreover, the A or aromatic acid amino acids, when juxtaposed to glycosylation sites in the tertiary fold, promoted protein folding along with the N-glycans during *N*-glycosylation (31, 33). In our study, presence of A amino acids at positions 3/−1/−2/−5 in combination with L at 2, indicates the preference of aromatic amino acids in the vicinity of the *N*-glycosylation site to promote protein folding via glycan-protein hydrophobic and nucleophilic interactions (Table 5; Figure 3). Interestingly, the same behavior of aromatic amino acids was suggested in the *N*-glycosylation process (37, 41, 43). In addition, our results also support the presence of polar acidic (N: Asp/Glu) residues preferably at positions 3/5/−4 (Table 5; Figure 2) which may help maintaining the solubility and ionic interactions of proteins.

In the first level of validation, the association patterns mined by MAPRes for *N*-glycosylated and non-*N*-glycosylated residues were determined by utilizing the NetNGlyc server. There were 226 potential sites for *N*-glycosylation (N+) found by NetNGlyc from the selected 15 proteins. The rest of the 642 Asn were considered to be N-residues. From the second level of validation, we selected the 438 experimentally defined glycopeptides from 130 reviewed proteins and found validation of MAPRes rules in all 438 glycoepitopes. We further modeled two cell membrane proteins, epidermal growth factor receptor (EGFR), and E-cad, because these proteins are heavily decorated with N-glycans and involved in cancers as a result of aberrant or excessive glycosylation (18, 31, 34, 44, 45). Inappropriate *N*-, and *O*-glycosylation often results in malfunction of EGFR—a validated oncogenic target in cancers, such as lung and breast (31, 44, 46–48). Actually, complete abrogation of EGFR *N*-glycosylation by tunicamycin led to increased susceptibility of EGFR to the tyrosine kinase (TK) inhibitor erlotinib, a frequently utilized drug to downregulate EGFR activation supporting constitutive proliferation of non-small cell lung cancer (49). This effect of *N*-glycosylation (a modification of the extracellular portion of EGFR) on the intracellular TK enzyme strongly suggests that the allosteric organization of EGFR TK is dependent on extracellular *N*-glycosylation events and that EGFR functions are indeed linked to the *N*-glycosylation status of EGFR.

In EGFR, 13 canonical *N*-glycosylation sites (N56, N73, N128, N175, N196, N352, N361, N413, N444, N528, N568, and N603) in its extracellular domain were reported in the UniProt database (44, 50–53). Amino acids 1–165 (domain I) and 310–480 (domain III) are involved in ligand binding, and domain II (165–309) constitutes the dimerization arm (46). These three domains, with domain IV (481–620) are essential for ligand binding and receptor dimerization/multimerization of the EGFR and result in the formation of an active EGFR. In this study, all 13 canonical motifs [N56 (NNCE), N73 (NYDL), N128 (NKTG), N175 (NMSM), N196 (NGSC), N352 (NATN), N361 (NCTS), N413 (NRTD), N444 (NITS), N528 (NVSR), N568 (NITC), and N603 (NNTL)] were found, validating the rules mined with MAPRes for *N*-glycosylation (Figure 3). In the first glycosylated motif NNCE, the presence of Cys at 2 indicates the involvement of NXCY sequon in *N*-glycosylation (44), and validates the respective MAPRes rules (<A,−1><L,2>;<P,−3><L,2>;<L,2><N,3>;<L,2><A,10>). In addition to the L amino acids at 2, N acidic amino acid at 3 and A-group at −1 positions cumulatively promote *N*-glycosylation in NNCE. The structural modeling of NNCE suggests that the aromatic R-group of phenylalanine at position −1 (in close proximity of N56) achieves the conformation favoring *N*-glycosylation of N56 (Figure 3). The same behavior of aromatic amino acids in the vicinity of Asn residues were proposed in the *N*-glycosylation process (41, 43). From our collective results for 13 EGFR *N*-glycosylated motifs, A residues were localized at positions +3/−1/−2, L at +2, N at 3/−4/5, and P at −3/9/10 position to support the *N*-glycosylation process. Most probably, the charge and polarity environments of these 13 motifs represent optimal environments for *N*-glycosylation, as the EGFR studied were isolated from proliferating cultured cancer cells.

Activation of EGFR depends upon its multimerization, and a model has been recently proposed whereby residues in the extracellular domain IV have been identified that promote multimerization and enhance intracellular TK activity (54). Although abolition of *N*-glycosylation at Asn 544 did not alter the TK activity of the mutant, whether inappropriately *N*-glycosylated sites in domain IV would do so as well has not been investigated. Multimerization of EGFR occurs in plasma membrane domains enriched in various glycoconjugates (55) and *N*-glycosylated membrane proteins have been identified that may control EGFR activity. For instance, *N*-glycosylated α5 integrin interacts with EGFR, promotes complex formation with α5β1 α6β4 heteropolymers and integrins, therefore, constitute proteins that may express mutated *N*-glycosylation sites in neoplastic diseases (56, 57).

E-cadherin, a calcium-dependent cell–cell adhesion molecule, is another key regulator of normal and neoplastic cells (18, 32, 34). The genome-wide association studies highlighted a strong correlation between *N*-acetylglucosaminyltransferase-III (GnT-III)-based structural modification of E-cad *N*-glycosylation in epithelial-to-mesenchymal transitions and susceptibility to colorectal cancer (18). In addition, the aberrant glycosylation on E-cad by GnT-V resulted in a poorer survival rate for gastric cancer patients (18, 34). E-cad contains four canonical significantly preferred extracellular *N*-glycosylation sites “N558 (NSTY), N570 (NGSP), N622 (NTSP), N637 (NWTI)” (45), supporting the mining rules of O amino acids at 1, L at 2, at −4, and A at 3, 5, and −10 position in *N*-glycosylation. In recent studies on site-directed mutagenesis in E-cad, the N558 (as Asn554), and N637 (as Asn633) were found aberrantly decorated with complex-type high-mannose N-glycans (β1,6 GlcNAc-branched), and critical for regulating the biological functions of E-cad in cancer (18, 34). For instance, the site-specific glycosylation at N558 elongate the deleterious branching structures and induces the tumor progression in gastric cancer (34). In our results, the presence of L amino acids at 2, N at −4, and A at 3, −5 collectively promote the site-specific glycosylation at N558 and direct the elongation of deleterious branching structures due to high electron density and polar environment which may induce the increased tumor progression in gastric cancer. In addition, the presence of N622 and N637 in close proximity of N558 (Figure 4) may further induce the elongation of glycan structures on E-cad.

**Figure 5:**
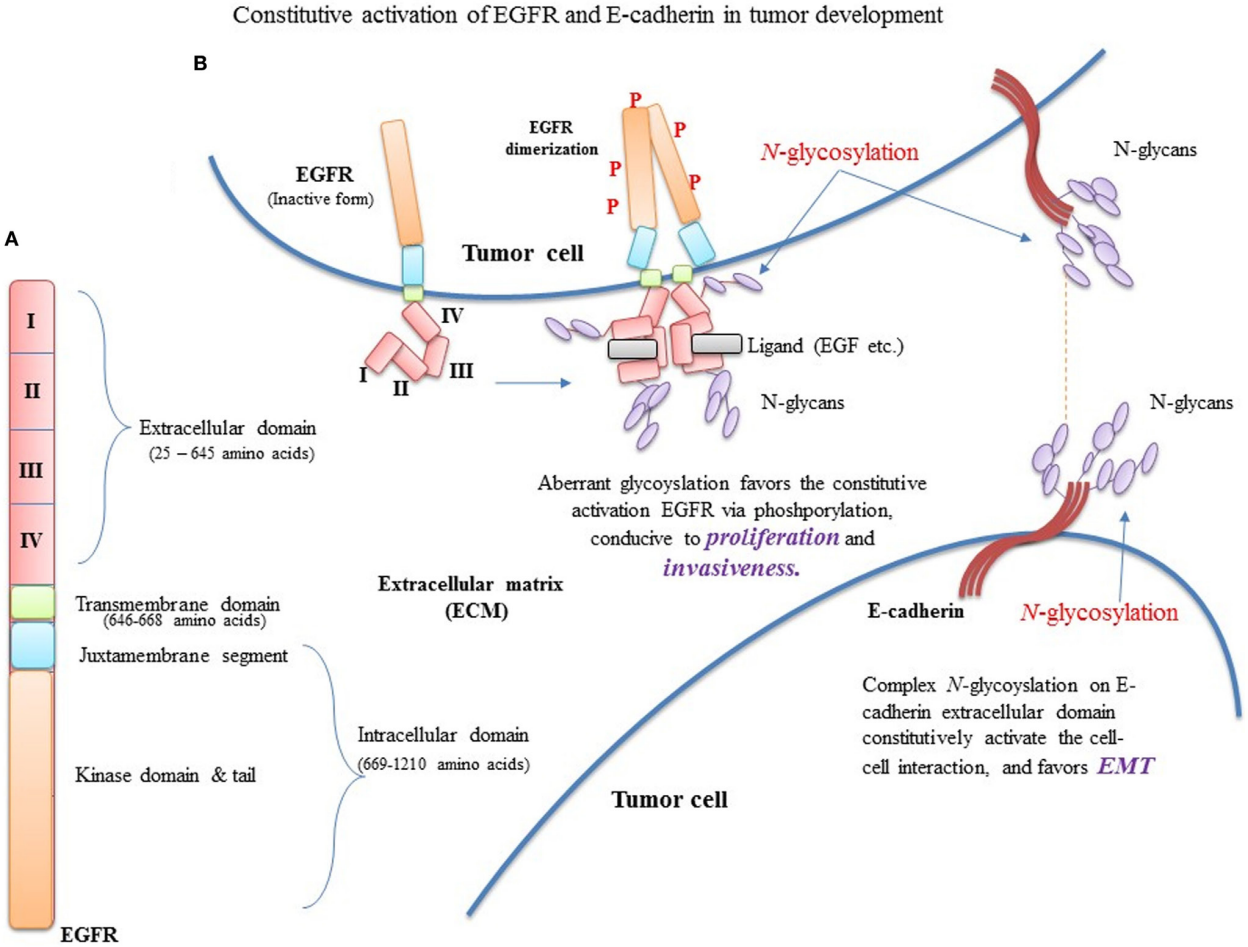
Constitutive activation of EGFR and E-cadherin (E-cad) in tumor development. **(A)** EGFR domain architecture in EGFR monomer. **(B)** Aberrant glycosylation favors the constitutive activation EGFR, conducive to proliferation and invasiveness. In addition, mutational changes in EGFR extracellular and transmembrane domains reduce phosphorylation, favoring proliferation.

Overall, the combination of polar and non-polar components, and acidic and basic groups of both penultimate and vicinal amino acids surrounding the *N*-glycosylation site, favor the normal *N*-glycosylation process. However, the mutational changes at *N*-glycosylation site and/or vicinal preferred sites may impact the oncogenicity of proteins (Figure 5) by modifying their polar chemistry.

### Conclusion and Future Prospects

Glycans remodel the protein backbone by reducing its conformational freedom and the loss of configurational entropy upon folding. To unravel the phenomenon of *N*-glycan remodeling in both ER and Golgi, charge and polarity of penultimate and vicinal amino acids were found important, and alteration in charge and polarity environment of Asn-containing epitopes results the heavy glycosylation (both on preferred and non-preferred sites) and reinforces the neoplastic phenotype. Thereby, the genetic alterations in many transmembrane receptors and adhesion proteins are important to determine the success or failure of glycosylation in these transmembrane proteins. This is particularly critical in neoplastic cells where disease-associated risks need to be assessed for many transmembrane oncogenic TKs.

In our analysis, cumulative affinity of both L amino acids at position 2 (with polar-uncharged R-groups) and O amino acids at position 1 (with non-polar aliphatic R-groups) was found significantly preferred for *N*-glycosylation. Moreover, remote parts of the protein chain rich in aromatic amino acids (A) support protein folding and promote the glycan–protein hydrophobic and nucleophilic interactions at positions 3/−5/−1 (Tables 3 and 4), in the presence of L amino acids at position 2, as in EGFR, TGFB1, and E-cad (Figures 3 and 4). The N amino acids at positions −4, 3, and 5 help maintaining the hydrophilic and other ionic interactions, as highlighted in EGFR and vitamin K-dependent protein C. Therefore, we suggest that the charge and polarity of R-groups in both penultimate and vicinal residues, notably the non-polar R-groups containing residues (O) at position 1, and polar R-groups containing L residues at position 2 along with the aromatic residues at positions 3/−1/−2/−5, acidic residues at 3/5/−4, and basic residues at −3/9/10 positions significantly contribute to normal *N*-glycosylation, and mutational alterations at these positions will significantly change the charge and polarity environment and cause constitutive activation of the glycoprotein by aberrant glycosylation leading to aggregation and intracellular phosphorylation in several carcinomas.

## Author Note

This publication is dedicated to the memory of Professor Dr. Nasir-ud-Din (1937–2016), founder and chairman of Institute of Molecular Sciences and Bioinformatics and Fellow of Pakistan Academy of Sciences.

## Author Contributions

MM designed the basic research theme, and contributed for data collection, analysis and validation. He also contributed significantly in manuscript writing. ZI defined and designed the research theme, methods, and generated various results. WQ contributed to generating results and participated in the improvement of the results interpretations. DH provided suggestions for improving the structure of the basic theme of the research and writing of the manuscript. He also helped to improve the scope of the study.

## Conflict of Interest Statement

The authors declare that the research was conducted in the absence of any commercial or financial relationships that could be construed as a potential conflict of interest.

## Footnotes

^1^http://www.cbs.dtu.dk/services/NetNGlyc/.

^2^http://www.uniprot.org/.

^3^http://dbptm.mbc.nctu.edu.tw/index.php.
